# Investigations to Evaluate Gastric Mucoadhesion of an Organic Product to Ameliorate Gastritis

**DOI:** 10.3390/pharmaceutics12040331

**Published:** 2020-04-07

**Authors:** Christina Winter, Sonja Hartl, Dagmar Kolb, Gerd Leitinger, Eva Roblegg

**Affiliations:** 1 Institute of Pharmaceutical Sciences, Pharmaceutical Technology and Biopharmacy, University of Graz, Universitätsplatz 1, 8010 Graz, Austria; christina.winter@uni-graz.at (C.W.); sonja.hartl@uni-graz.at (S.H.); 2 Core Facility Ultrastructure Analysis, Center for Medical Research, Gottfried Schatz Research Center, Medical University of Graz, Neue Stiftingtalstrasse 6/II, 8010 Graz, Austria; dagmar.kolb@medunigraz.at; 3 Division of Cell Biology, Histology and Embryology, Gottfried Schatz Research Center, Medical University of Graz, Neue Stiftingtalstrasse 6/II, 8010 Graz, Austria; gerd.leitinger@medunigraz.at; 4 Research Center Pharmaceutical Engineering GmbH, Inffeldgasse 13, 8010 Graz, Austria

**Keywords:** mucoadhesion, organic product, rheology, wettability, falling liquid film technique, in vitro studies, ex vivo studies

## Abstract

Gastritis is an inflammatory disease leading to abdominal pain, nausea, and diarrhea. While therapy depends on etiology, adhesive agents protecting the gastric tissue represent a promising treatment option. Caricol^®^-Gastro is an organic product that significantly decreased gastritic abdominal pain in a recent clinical study. To investigate whether this beneficial effect can be attributed to the formation of a protective layer covering the gastric mucosa after oral application, several methods were used to determine adhesion. These include macro-rheological measurements and gastric mucin interactions, which were correlated to network formation, examined by Cryo-scanning electron microscopy technique, wettability via sessile drop method on human gastric adenocarcinoma cell layers, and ex vivo adhesion studies on gastric porcine tissue with the falling liquid film technique considering physiological conditions and Franz diffusion cells for quantification. The results showed that Caricol^®^-Gastro formed a stable viscoelastic network with shear thinning properties. It exhibited high wettability and spreadability and adhered to the excised gastric mucosa. We found that oat flour, as the main ingredient of Caricol^®^-Gastro, supports the gel network regarding viscoelasticity and, to a lesser extent, adhesion in a concentration dependent manner. Moreover, our data highlight that a variety of coordinated methods are required to investigate gastric adhesion.

## 1. Introduction

Gastritis is an inflammatory disease that affects the lining of the mucosa in the stomach. In principle, a distinction can be made between two forms of gastritis, i.e., acute and chronic gastritis. Acute gastritis occurs suddenly and can be caused by stress, medication intake (e.g., non-steroidal anti-inflammatory drugs or steroids), and alcohol or nicotine intoxication [1,2]. Due to damage to the mucosal defenses, surface necrosis takes place, which leads to symptoms, such as pain, loss of appetite, or nausea [3]. Chronic gastritis develops gradually over months and appears either as non-atrophic or atrophic form [4,5,6,7]. The atrophic form involves the loss of gastric glandular cells, which leads to a disorder in secretion of essential substances and consequently digestive problems [8]. It is mostly caused by infections, mainly by *Helicobacter pylori*, but can also have an autoimmune origin [3,5]. Other causes include infections by organisms, such as *Mycobacterium avium* intracellular, *Herpes simplex*, and *Cytomegalovirus,* bile acid reflux, radiation therapy and others. Thereby, symptoms include pain in the upper abdomen, bloating, nausea, vomiting, and diarrhea [8,9].

Treatment of gastritis depends on the etiology. Infectious chronic gastritis is treated with specific antibiotics combined with proton-pump inhibitors, and autoimmune gastritis is managed with iron and vitamin B 12 substitutes [10,11,12]. Other forms of alleviation of gastritis include cessation of stress, medications, alcohol and nicotine, and spicy food [9,11,13]. Additionally, cytoprotective and mucoprotective agents can be used that protect the injured lining mucosa in the stomach and support standard medical treatment [1,12]. Caricol^®^-Gastro is an organic product that has been recently investigated in a randomized, double blind placebo controlled clinical trial regarding its effect on patients diagnosed with chronic gastritis. The results showed that the pain load associated with chronic gastritis was significantly reduced in the patient group treated with Caricol^®^-Gastro [14]. Caricol^®^-Gastro is available as gel (packed into sticks) and consists mainly of two natural components, i.e., papaya pulp and oat flour. Papaya contains a variety of enzymes, such as papain, chymopapain, and lysozyme, Vitamin C, antioxidants, bioflavonoids, and minerals, which are responsible for a beneficial nutritive effect [14,15,16,17]. Clinical observations showed that in patients with gastrointestinal disease papaya interacted with reactive oxygen species and reduced the secretion of gastric acid caused by histamine [17,18]. Oat from *Avena Sativa L.* comprises fatty acids, antioxidants, vitamins (E and B), minerals, and cell wall polysaccharides such as starch and β-glucan. [19,20]. The cell wall polysaccharides consist of 9–28% β-glucan and 40–60% starch, whereas the amylose and amylopectin content in oat starch is about 25% and 75%, respectively [21,22]. Studies show that β-glucan fibers contribute most to the viscosity and wetting ability of oat preparations [22,23,24]. These fibers form a mucous gel upon contact with water that is supposed to adhere to the moist surface lining in the stomach and to act as a protective layer. The adhesion between two materials, at least one of which is a mucous membrane surface, is also called mucoadhesion. In the field of drug formulation, mucoadhesion is an important characteristic for prolonging the retention time of dosage forms on different mucous membranes, such as the gastrointestinal tract, vagina, lung, and oral mucosa. On the one hand, the absorption of drugs can be increased; thus, improving their bioavailability, and on the other hand, (injured) tissues can be moistened or protected. The process of mucoadhesion is divided into two stages, i.e., the contact and the consolidation stage [25]. The contact stage is defined as intimate contact (wetting) between the polymer and the mucous membrane, while the consolidation stage encompasses the various physicochemical interactions leading to prolonged adhesion [26,27]. There are six theories that govern the interactions and mechanisms of mucoadhesion, including (i) wetting, (ii) dehydration, (iii) diffusion, (iv) adsorption, (v) electronic, and (vi) mechanical theories [25,26,28,29,30,31,32,33]. Moreover, the extent of adhesion of a particular polymer depends on various factors such as molecular weight, flexibility of the polymeric chains, water retention capacity, viscoelastic behavior, and ionic state of the polymer. However, these properties are dependent on the physiological pH of the respective mucosal area and the polymer concentration and should, thus, be taken into account [25,28,34]. In vivo methods to determine mucoadhesion in the gastrointestinal tract are scarce and include e.g., gamma scintigraphy, the usage of radioisotopes/x-ray, or the tension resistance method [35,36,37]. However, these methods often show incoherence among the results due to different parameters and conditions. Moreover, in vivo studies are expensive, time-consuming, and require ethical approval [38]. As an alternative, in vitro examinations can be carried out to predict the behavior of a formulation under physiological conditions and to improve the understanding of underlying mechanisms of action. This would reduce costs incurred by clinical studies, enable a faster screening of dosage forms, and facilitate rational formulation design or—if necessary—formulation improvement [39,40]. However, for the characterization of mucoadhesive effects, there is no universally valid in vitro model available Therefore, it is hypothesized that a combination of coordinated methods has to be applied. The most frequently used in vitro methods include rheological techniques, surface plasmon resonance, isothermal titration calorimetry, tensile strength test, shear strength test, peel strength test, and contact angle measurements performed on specific surfaces [28,34,41]. In addition, mucoadhesion can be tested ex vivo using excised tissues [38,39,41,42]. Thereby, the everted gut sac technique, atomic force microscopy and the falling liquid film method are the most frequently used techniques to study mucoadhesion along the oro-gastrointestinal tract [43,44,45].

Since the efficacy of Caricol^®^-Gastro to alleviate abdominal pain and reduce constipation and bloating has been proven in clinical studies, it is obvious that it forms a protective layer over the gastric mucosa due to its mucoadhesive properties [14,16]. Therefore, to be able to correlate clinical effectiveness with in vitro performance, a suitable mucoadhesion tool was built and Caricol^®^-Gastro was used as model substance. To this end, the viscoelastic behavior and the gel-like network formation of the agent was investigated taking into account physiological factors, such as the production of gastric juice, gastric motility, physiological body temperature (37 °C) and gastric mucin interaction [46]. In order to evaluate whether adhesive properties can be attributed to the oat flour only, additional investigations were carried out with pure oat flour formulations soaked and heated in water considering the influence of the polymer’s concentration. Moreover, wettability studies were performed on human gastric adenocarcinoma (AGS) cell-coated surfaces considering horizontal and vertical (35°) positions to properly mimic physiological conditions in the stomach. Excised gastric porcine mucosa was used with the falling liquid film technique to macroscopically study the adhesion behavior of the organic product on physiologically relevant tissues [47]. In order to be able to carry out quantification, all formulations were stained with methylene blue and applied to cut pieces of excised gastric porcine mucosa. The mucosa was placed into agitated Franz diffusion cells to ensure controlled conditions during the experiment. Finally, the amount of stained formulation washed away over time was measured spectrophotometrically [48].

## 2. Materials and Methods

### 2.1. Preparation of the Investigated Formulations

The main ingredients of the studied formulation (i.e., commercially available Caricol^®^-Gastro) are summarized in Table 1. The preparation of Caricol^®^-Gastro is described in patent EP 3131565 A2. Briefly, oat flour is dispersed in water, heated to 70–90 °C, and stirred in a closed system for at least 30 min. Subsequently, papaya pulp, natural flavoring, and apple concentrate, to adjust the pH to 4.5–5.5, are added, cooled and filled into sticks. The oat extracts used in this study were produced from oat flour powder as aqueous decocts, according to a modified procedure described in European Pharmacopoeia (Ph. Eur. 9.1.4.3, 23a). For this purpose, two extracts were prepared to study the influence of the concentration on the network formation taking into account the stirring time. First, 6.25 g (*w/w*) of the oat powder (according to recipe specification) were dispersed in MQ-water to 100 g, heated to 100 °C, stirred for 30 and 60 min at 250 rpm in a closed vial, respectively, and cooled to room temperature (RT, referred to as oat I). Second, to consider the other semi-solid and liquid ingredients of Caricol^®^-Gastro (i.e., papaya pulp, apple juice concentrate, and residual water), the oat powder concentration was adjusted accordingly to 14.29 g (*w/w*). Processing was carried out using the same method as described above (referred to as oat II).

### 2.2. Macrorheological Measurements Taking into Account Interactions with Simulated Gastric Fluid and Gastric Mucins

Rheological studies were conducted with Caricol^®^-Gastro, oat I and oat II. To prevent liquid evaporation a built-in evaporation protection hood was used for all measurements. The linear viscoelastic region (LVE) was determined via frequency sweep between 0.1 and 100 Hz and amplitude sweep at a constant angular frequency of 0.1 rad/s and deformation (γ) between 0.1 to 100. The viscoelastic moduli G’ (i.e., elastic modulus) and G’’ (i.e., viscous modulus) were determined via strain-controlled oscillation using a Physica MCR 301 rheometer (Anton Paar) with a CP-50-1 measurement system (cone-plate geometry) at RT (25 ± 0.5 °C) and 37 ± 0.5 °C [42]. The loss factor tanδ, which describes the ratio of viscous and elastic behavior, was calculated according to G’’/G’ for each measurement point. Viscosity η was determined using the same measurement system with shear rate-controlled rotation at both 25 ± 0.5 °C and 37 ± 0.5 °C. The applied shear strain for both experiments were angular frequencies between 0.1 and 100 1/s to consider naturally occurring strain due to gastric motility [43].

To consider interactions with gastric fluid, Caricol^®^-Gastro, oat I and oat II were mixed with simulated gastric fluid (SGF) in the ratio 1:1. SGF was prepared according to the United States Pharmacopoeia [49]. Briefly, 2.06 g NaCl were dissolved in 1000 g MQ-water and the pH was adjusted to pH 1.2. The viscoelastic properties were determined at 37 °C using the same test setup as the pure formulations. The results of both measurements were compared.

To investigate interactions between porcine stomach mucin and Caricol^®^-Gastro, oat I and oat II respectively, rheological studies were carried out. To this end, lyophilized mucin from porcine stomach (Sigma Aldrich, Munich, Germany, Type III, partially purified powder) was dispersed in 0.1 M HCl (10 mg/mL). [50,51]. Subsequently, the mucin solution was mixed with each formulation to a final mucin concentration of 5 mg/mL and adjusted. The viscoelastic properties were determined at 37 °C with the same test setup as before and used to calculate interaction parameters according to Rossi et al. [46,52,53]. Briefly, the viscoelastic parameters were calculated as differential values to assess Δ η/η, ΔG’/G’ and Δtan δ as summarized in Table 2. To consider shear strain, the value at the lowest angular frequency (0.1 s^−1^), referred to as low in the graph, medium angular frequency (5.18 s^−1^), referred to as medium in the graph, an highest angular frequency (100 s^−1^), referred to as high in the graph were considered for calculation.

### 2.3. Cryo-Scanning Electron Microscopy (Cryo-SEM) Preparation

To confirm the network formation evaluated from rheological studies, the microstructure of Caricol^®^-Gastro, oat I and oat II were visualized using the Cryo-SEM technique (Quorum PP3010T). Prior to visualization the samples were frozen under slush liquid nitrogen and transferred with a vacuum transfer device into the preparation chamber for subsequent processing and observation. The cryo-preparation chamber was connected to a GEMINI Sigma 500 (ZEISS Company, Oberkochen, Germany) SEM, which included a nitrogen gas cold stage. In the chamber the samples were fractured, sublimated, and sputter coated with palladium. The fractured material was transferred into the SEM specimen chamber before image recording.

### 2.4. Calculation of the Pore-Size Distribution

The pore size distributions were calculated using ImageJ-Fiji software package. To this end, the SEM-images were converted into binary files using the threshold function. From the inverted binary files, the Feret diameters of at least 500 pores were calculated. For the determination of the pore-size, each pore was assumed as a particle and calculated according to Equation (1), where the pore is defined as the volume of a particle (V) and d is the Feret diameter:

(1) $$V=\frac{4}{3}\times \pi \times {\left(\frac{d}{2}\right)}^{3}$$

The pore–size distribution was then calculated as volume percentage to consider large and small pores.

### 2.5. In Vitro Wettability Studies

#### 2.5.1. AGS-Cell Cultivation

Human gastric adenocarcinoma cells (AGS) were kindly provided by B. Rinner (Medical University Graz, Austria) and cultivated in high glucose Dulbecco’s modified Eagle medium (DMEM) supplemented with 2 mM l-Glutamine and 10% fetal bovine serum (FBS) purchased from Gibco, Life Technology Corporation (Painsley, UK). Culture conditions were kept constant at 37 °C in a humid 95% air/5% CO_2_ atmosphere. Routinely, medium was changed every other day and AGS-cells were sub-cultivated at 80% confluence with 0.25% trypsin-EDTA at a split ratio of 1:6. Prior to use, cells were cultured on glass coverslips (Ø 25 mm) at a concentration of 1.5 × 10^5^ cells/slip. After careful microscopic verification of the formation of a confluent cell layer, cells were further treated for contact angle studies, according to previously published protocols [44,45,46]. Briefly, the cover slips were washed with phosphate buffered saline (PBS, pH 7.4, Gibco, Life Technologies Corporation, Painsley, UK) and fixed in a neutral buffered 10% formalin solution for 15 min (Sigma-Aldrich^®^, Munich, Germany). The cells were further dehydrated in an ascending alcohol (EtOH) series, i.e., 50%, 70%, 96%, and 100% alcohol (i.e., ethanol, EtOH, analytical grade, VWR, Vienna, Austria) series for 30 s each to minimize shrinkage effects resulting in inhomogeneous surfaces. To consider dilution of the formulation with gastric juice, Caricol^®^-Gastro was mixed with 0.1 M HCl in the ratio 1:1 prior to the contact angle measurements.

#### 2.5.2. Contact Angle Measurements

**Figure 1:**
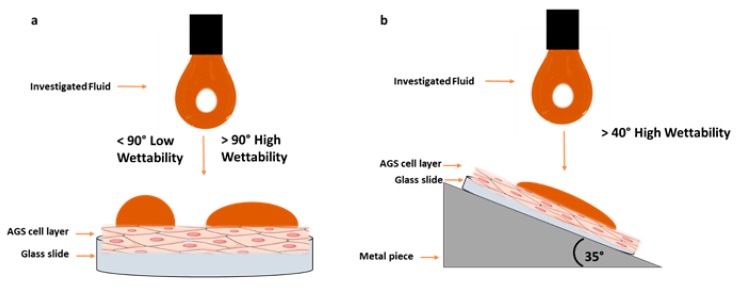
Schematic illustration of the experimental wettability set-up performed at horizontal (**a**) and 35° (**b**) positions.

The prepared cell surfaces were utilized to determine wettability effects via contact angle measurements at the liquid/semisolid interface. Therefore, the glass slides with the fixed cells were placed horizontally and in a 35° angle by gluing the slide on a metal piece (60 × 45 mm, 35° slope). Each measurement was carried out with the sessile drop method at 22 °C with a drop volume of 1 µL. Images of the liquid droplets that settled on the cell layers were captured immediately after the drop wetted the surface. Contact angles were analyzed with the standard software using the Young equation (Equation (2))

(2) $$\gamma_{LG}\times \cos\theta =\gamma_{SG}-\gamma_{SL}$$

where, γ_SL_, and γ_SG_ are the corresponding surface energies of liquid solid interface per unit area [54,55]. All investigations were carried out six times (*n* = 6). A MQ-HCl mixture (resulting in 0.05 M HCl) was used as control. A schematic illustration of the wetting experiments is shown in Figure 1.

### 2.6. Ex Vivo Studies with the Falling Lquid Film Technique

Mucoadhesion of Caricol^®^-Gastro was investigated using gastric porcine excised mucosa on a modified falling liquid film technique as previously described by Gradauer et al. [39]. For this, porcine stomach was obtained from freshly sacrificed pigs (age < 6 months; Marcher Fleischwerke, Graz, Austria) and transported in 4 °C Krebs buffer (Sigma-Aldrich^®^, Munich, Germany) to the laboratory. The gastric mucosa was cut from the grater curvature into 20–30 cm^2^ pieces, carefully rinsed with Krebs buffer and mounted with the mucosal side up on a semicylindrical Plexiglas^®^ tube at a 35° angle. The equipment was put on a shaker, agitated at 100 rpm to mimic gastric motility [47]. A peristaltic pump was used to constantly flush the mucosa with 0.1 M HCl (pump speed of 2 mL/min), simulating the gastric fluid in the stomach. For visualization purpose, 2 g of Caricol^®^-Gastro were died with 5 mg of methylene blue and directly applied on the top of the tube to mimic the transport through the esophagus and the gastric fold to the pyloric sphincter. The amount of remaining material was macroscopically investigated over a time period of 30 min and compared to 2 g organic oat I and oat II dyed with 5 mg of methylene blue and 2 g MQ-water dyed with 5 mg of methylene blue (control).

#### Ex Vivo Quantification with Franz Diffusion Cells

To quantify the amount adhering to the excised gastric tissue, 0.5 g of each formulation stained with methylene blue (10 µL, concentration: 1 mg/mL) was applied to adequate sections (approximately 2 × 2 cm) of the gastric porcine mucosa. MQ water dyed with methylene blue was used as control. The mucosa sections were placed into static Franz diffusion cells and each donor compartment was filled with 1 mL of 0.1 M HCl. The whole equipment was placed in an incubator at 37 °C and agitated at 50 rpm. After 1, 5, 20, 30, and 60 min samples were withdrawn from the donor compartment and refilled with fresh media. Subsequently, the samples were centrifuged at 14,000 rpm and the methylene blue concentration in the supernatant was determined spectrophotometrically using a nano-photometer (Implen, Munich, Germany) via single wavelength measurement at 665 nm [43].

### 2.7. Statistical Analysis

Depending on the respective method, three- and six-fold determinations were carried out. The results are presented as the mean values ± standard deviation (SD). To evaluate statistical significance between Caricol^®^-Gastro, oat I, oat II and control (MQ-water), one-way analysis of variance (ANOVA) was used. Differences were considered to be significant at a level of *p* < 0.05 (*), *p* < 0.01 (**), and *p* < 0.001 (***).

## 3. Results

### 3.1. Macroscopical Evaluation of the Extracts

After the 30 min of stirring and heating according to Ph. Eur. 9.1.4.3, 23a, no complete swelling of the oat powder could be achieved resulting in an inhomogeneous dispersion. After increasing the stirring time to 60 min, a homogenous swollen dispersion was obtained.

### 3.2. Rheological Analysis

**Figure 2:**
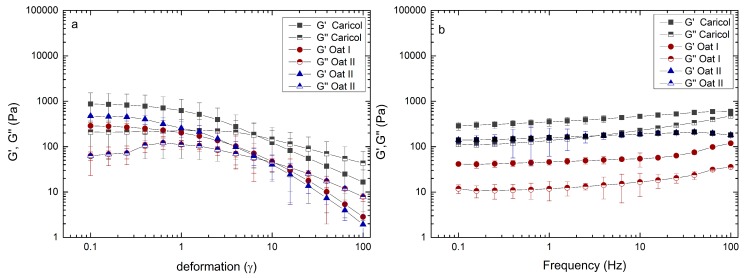
Determination of the linear viscoelastic region (LVE) determined via amplitude sweep (**a**) and frequency sweep (**b**).

**Figure 3:**
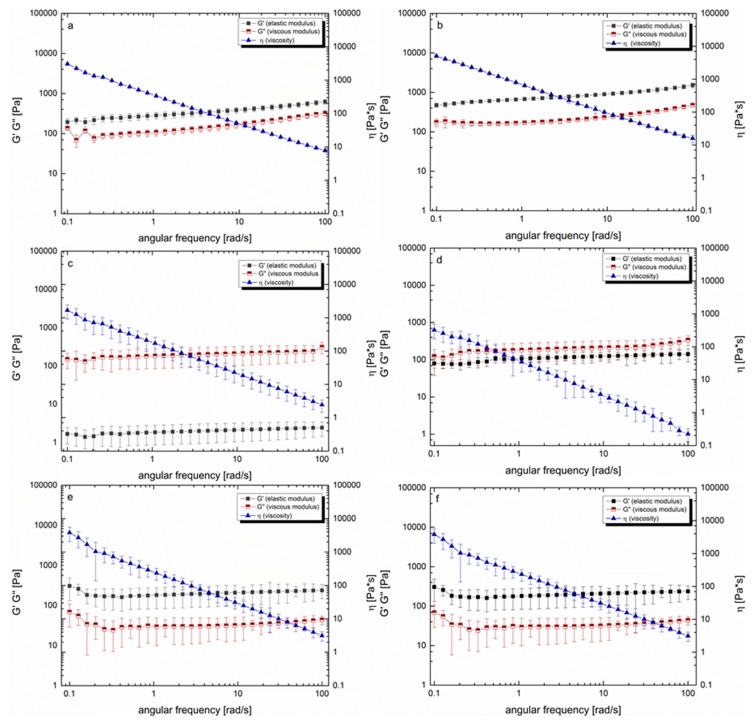
Rheological investigations of Caricol^®^-Gastro at room temperature (RT) (**a**) and 37 °C (**b**), oat I (6.25 wt %) at RT (**c**) and 37 °C (**d**), and oat II (14.29 wt %) at RT (**e**) and 37 °C (**f**).

Prior to the oscillation measurements, the LVE region of each formulation was determined at 37 °C. Both amplitude and frequency sweep were carried out to ensure that the strain response of the formulations was proportional to the input strain amplitude. The results showed that the measurement range for deformation (γ = 1) and the frequency between 0.1 to 100 Hz, which equals 6.28 to 628.31 rad/s, were within the LVE region (Figure 2a,b). The viscoelastic characteristics for Caricol^®^-Gastro showed that the elastic modulus was larger than the viscous modulus (G‘ > G‘‘). This suggests the formation of a crosslinked network. This network remained stable over a wide range of shear rates (0.1–100 rad/s). The loss factor tan δ decreased slightly from 0.7 at low shear rates (0.01–0.4 rad/s) to 0.5 at higher shear rates (>0.5 rad/s), which classifies Caricol^®^-Gastro as a strongly crosslinked viscoelastic fluid. The formulation exhibited the behavior of a non-Newtonian, shear-thinning fluid. While the dynamic viscosity changed as a function of the applied shear stress, alterations of the temperature, i.e., from RT to body temperature, did not impact the flow behavior of Caricol^®^-Gastro (Figure 3a,b). Oat I with a concentration of 6.25 wt% and stirred for one hour at elevated temperature did not show the characteristics of a crosslinked fluid (Figure 3c). Contrary to Caricol^®^-Gastro it was found that the viscous modulus dominated the elastic one, which significantly increased the tan δ to values > 25 ***. With increasing temperature, the elastic modulus increased and the tan δ values decreased to 1.2–1.6 *** (Figure 3d). However still the ratio of moduli did not change. The standard deviations of all measuring points were high, indicating an inhomogeneous mixture. By increasing the oat flour concentration to 14.29 wt%, which represents the concentration under real conditions, the ratio of the viscoelastic moduli changed at RT and 37 °C (Figure 3e,f). The elastic modulus was larger than the viscous one and the tan δ value was < 1. Oat II exhibited a shear-thinning behavior and displayed the characteristics of a crosslinked viscoelastic fluid (G ‘> G‘‘), which was comparable to Caricol^®^-Gastro. The loss factor tan δ remained in a constant low range (i.e., 0.3–0.2) over the applied shear stress.

**Figure 4:**
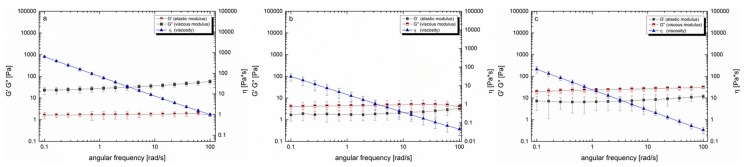
Rheological investigations of Caricol^®^-Gastro with simulated gastric fluid (SGF) (**a**), oat I with SGF (**b**), oat II with SGF (**c**) at 37 °C.

After considering the residual gastric juice, it was found that due to the dilution step, the viscosity and viscoelastic moduli decreased in all three formulations. However, the moduli G’ and G’’ remained in the same ratio as in the pure formulations (Figure 4a–c). Investigations regarding the mucin interactions showed that the elastic modulus dominated the viscous modulus over the entire shear rate. The loss factor tan δ remained nearly constant, i.e., 0.71 and 0.84 (data not shown). The viscosity, measured at 37 °C, ranged from 124 Pa·s at low shear rates to 0.1 Pa·s at the highest shear rate applied, exhibiting a shear thinning behavior. The addition of mucin to the formulations slightly increased the viscosity; however, the ratio of the viscoelastic moduli remained unchanged.

**Figure 5:**
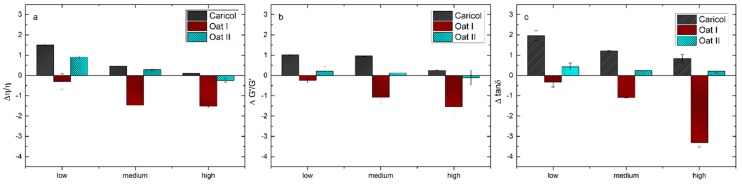
Rheological parameters for Δ η/η (**a**), ΔG’/G’ (**b**), Δtanδ (**c**) for Caricol^®^-Gastro, oat I, oat II in the presence of porcine gastric mucin calculated as differential interaction parameters [46].

The differential parameters of the viscosity, elastic moduli and tan δ were calculated by subtracting the sum obtained from the polymer solution and mucin solution from the value observed for the polymer-mucin mixture (Figure 5a–c). According to the literature, values higher than zero indicate the formation of mucoadhesive joints between the mucus solution and the polymer formulation [46]. In this study, the highest positive value was observed for Caricol^®^-Gastro at low shear rates, indicating a mucoadhesive interaction. With increasing shear rates, the values decreased towards zero, which shows that the interactions became weaker with increasing shear strain. For oat I, we observed negative values at all three shear rates used for calculations. At higher shear rates, the values decreased even further, indicating that no adhesive interaction between mucin and oat was established. Oat II, which had a higher concentration, showed positive values for the viscosity at low and medium shear rates. The values calculated for the elastic moduli were only slightly positive, and again, the interactions became weaker with increasing shear strain until they were close to zero.

### 3.3. Microstructure and Pore Size Distribution

**Figure 6:**
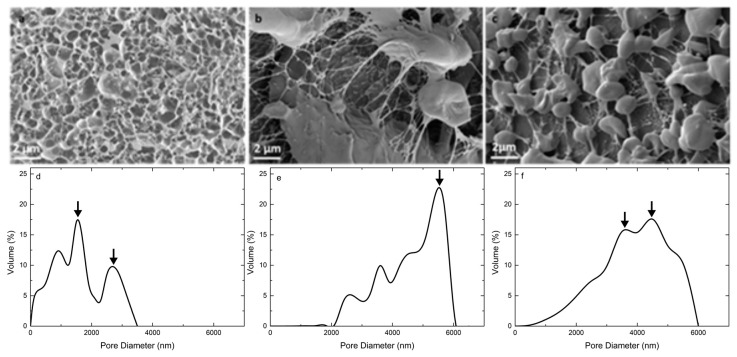
Representative Cryo-SEM micrographs of Caricol^®^-Gastro (**a**), oat I (**b**) and oat II (**c**). The diagrams show the pore size distribution of Caricol^®^-Gastro (**c**), oat I (**d**) and oat II (**f**).

Cryo-SEM images revealed a stable and coherent network structure for Caricol^®^-Gastro with heterogeneous pore sizes. The pore size distribution ranged from 50 to 3200 nm, with the highest volume percentage at 900 and 2700 nm (Figure 6a,d). Oat I also showed a network-like structure interspersed with starch particles, which exhibited sizes from 5 up to 60 µm. However, the pores were much larger displaying their highest volume percentage at 5800 nm. Furthermore, the fibers appeared thinner and consequently more delicate compared to Caricol^®^-Gastro (Figure 6b,e), which exhibited thick and tight network fibers. For oat II we observed more homogeneously dispersed starch particles and a tighter network structure. Below the range of 2000 nm a higher volume percentage of pores was observed compared to oat I (almost no pores). The volume percentage of pores larger than 5000 nm decreased compared to oat I and the maximum volume percentage was between 3600 and 4800 nm (Figure 6c,f).

### 3.4. In Vitro Wettability Studies

The contact angles for Caricol^®^-Gastro were determined after mixing of the sample with 0.1 M HCl to consider gastric juice in the stomach. On the horizontally arranged cell layer a contact angle of 38.4° ± 4.5° was observed. Due to 35° inclination of the cell layer, the values decreased to 28.0° ± 0.8° (*p* < 0.01). For control, MQ- water was mixed with HCl. The contact angle on AGS-cells was 101.1 ± 2.0 in the horizontal mode and decreased significantly (*p* < 0.001) in inclined position. Due to agglutination in the syringe it was not possible to determine the contact angle for oat I and II. All values are summarized in Table 3.

### 3.5. Ex Vivo Falling Liquid Studies

Ex vivo adhesion studies of Caricol^®^-Gastro, oat I, oat II and MQ-water were carried out on excised porcine stomach mucosa using the falling liquid film apparatus. To this end, all formulations were stained with methylene blue in order to perform a precise macroscopic evaluation of the adhesion effect. The results showed that for Caricol^®^-Gastro most of the initially applied material remained on the mucosa for more than 30 min. Neither the rinsing with HCl during the experiments, to simulate the production of gastric acid, nor the motility mimicked by agitation influenced the layer formation. For oat II similar results were obtained. After 30 min oat II was partly washed away; most of the material was still found in the gastric folds. However, for oat I and MQ-water most of the initially applied material (i.e., 2 g each) was flushed away within the first 10 min. For oat I and MQ-water only a thin blue-dyed layer remained.

#### Ex Vivo Quantification via Franz Diffusion Cells

**Figure 7:**
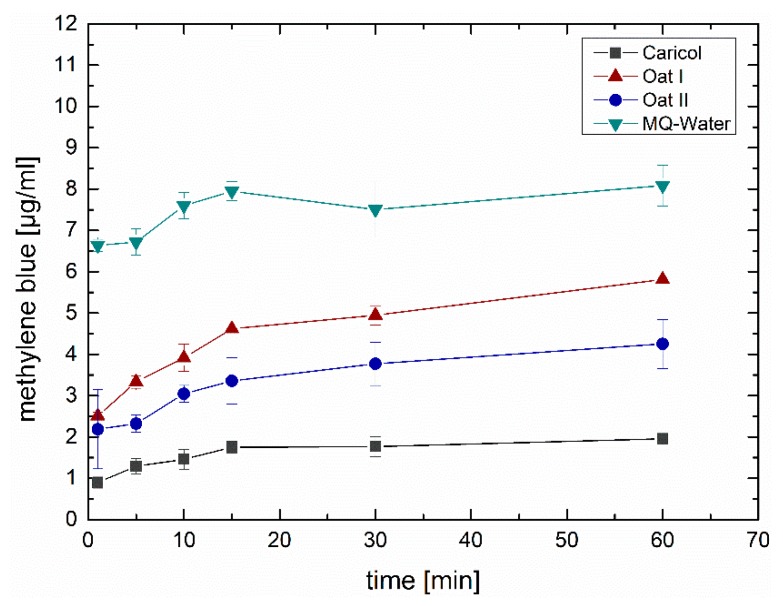
Quantification of methylene blue contained in the formulations (Caricol^®^-Gastro, oat I, oat II and MQ-water) that was washed away from the porcine gastric mucosa over time.

Investigations to quantify the adhesive portion of the respective formulations tested showed that only small amounts of the formulation were washed away with Caricol^®^-Gastro. There was hardly any increase in methylene blue, which suggests that most of the methylene blue contained in the formulation remained on the mucosa for a period of 60 min (Figure 7). This good adhesion behavior correlates with the investigations with the falling liquid technique. Furthermore, the results showed that oat II was washed away more slowly than oat I. However, in both formulations, significantly (*p* < 0.01) less methylene blue was washed away compared to the control. For MQ-water, which served as control, most of the methylene blue was washed off the surface within the first minute after application.

## 4. Discussion

From recently performed clinical studies it is obvious that Caricol^®^-Gastro shows adhesive properties to the gastric mucosa due to its pain-reliving effects in the clinical course of chronic gastritis [14]. To be able to build up a sound understanding of the mechanisms behind adhesive effects in a cost-effective and time-saving manner, related in vitro mucoadhesive test methods that take into account the physiological conditions of the respective barrier are highly important. Moreover, the establishment of such a tool would also allow to predict performances of other mucoadhesive dosage forms and facilitate rational formulation design. Thus, a combination of different methods, i.e., rheological methods, visualization of the network by SEM, in vitro wettability studies using the sessile drop method, and ex vivo studies conducted with the falling liquid film method were used and adjusted where necessary to investigate the mechanisms of adhesion of the model formulation Caricol^®^-Gastro. Quantification of the material that was washed off the gastric mucosa was performed with Franz diffusion cells using methylene blue as staining agent. To examine whether the adhesive effects were mainly due to oat flour, we investigated pure oat preparations at different concentrations.

According to literature, the most valid rheological method to predict the deformation and flow behavior of substances and allows to draw conclusions on the adhesive properties, is to determine the viscoelastic characteristics via oscillatory measurements [43]. Thereby, viscoelastic materials are characterized by a delayed reaction to physiological occurring shear strain and relaxation. This behavior can depend on the degree of shear stress, shear time and temperature available at the respective biological site of interest [43,48,49]. To simulate physiological conditions in the stomach, we applied increasing steady shear stress up to 100 rad/s at 37 °C adapted from Feng et al., who recently investigated the in vitro digestion process of oat products [50]. For Caricol^®^-Gastro, it was found that the dynamic viscosity decreased dependent on the applied shear stress, indicating a shear thinning behavior. On the one hand, the temperature (i.e., 25 °C versus 37 °C) did not impact the flow behavior of Caricol^®^-Gastro. This is of paramount importance as it ensures that the gel does not liquefy in the body. However, on the other hand the shear-thinning properties are necessary for Caricol^®^-Gastro to enter the stomach after oral application [16,24,46]. The viscoelastic properties of the product revealed a coherent and stable network under physiological shear stress as the elastic modulus dominated the viscous one [48,51]. Thereby, disulfide bridges were formed between the cysteine-rich domains and the β-glycans, which created a cross-linked three-dimensional gel, that was further strengthened by hydrogen bonds [56]. These results were further confirmed by Cryo-SEM. The micrographs of Caricol^®^-Gastro showed a tight and coherent network with a maximum pore size diameter of 3200 nm. This suggests that, considering the aforementioned shear thinning properties, Caricol^®^-Gastro is able to spread evenly over the mucous membrane and coat it. Mixing with SGF did not change the viscoelastic moduli and shear thinning behavior, but only shifted the data towards lower values, thus, dilution with gastric juice can be neglected. The in vitro wettability studies correlated with the obtained data and showed that Caricol^®^-Gastro resulted in a low contact angle, indicating high spreadability and consequently high wettability [45]. According to literature, contact angles below 90° show increased spreadability of an investigated material on specific surfaces [34,44,54]. The contact angle for Caricol^®^-Gastro decreased significantly (*p* < 0.01) from horizontal to inclined position (38° ± 5° vs. 28° ± 1° **) [37,44,45]. The dilution to account for residual gastric juice did not affect the high wettability of Caricol^®^-Gastro [55]. Contact angles for the pure (undiluted) Caricol^®^-Gastro formulation were in the same low range (data not shown). The adhesive interactions with gastric mucus were strongest for Caricol^®^-Gastro, which is consistent with the results from the rheological studies and the visualized network structure. The contact between the polymeric compounds of Caricol^®^-Gastro, or more precisely β-glycan and α-(1→4)-d-galacturonan of pectin and the mucins led to physical entanglement [57,58]. Subsequently, chemical bonds, such as hydrogen bonds or ionic interactions between the mucins and the polymeric compounds were formed. These interactions decreased with increasing shear stress, which is consistent with the literature concerning other polymeric compounds [46,47,48]. This assumption was confirmed by the ex vivo experiments. To this purpose, excised gastric pig mucosa was used because of its similarity to human gastric tissue in terms of, e.g., histological and physiological features [56]. For an accurate macroscopic evaluation, all formulations tested were labeled with methylene blue [55,57]. For comparison purpose, the 35° angle used for the contact angle measurements was also chosen for the falling liquid film apparatus. Moreover, gastric fluid production was considered by flushing the tissue with HCl and gastric motility was taken into account by shaking the apparatus at 50 rpm during the entire experiment [47]. The results showed that Caricol^®^-Gastro adhered effectively to the gastric mucosa supporting the in vivo data [14]. After 30 min, most of the applied formulation was still in place, which can be attributed to the viscoelastic properties, the stable network formation and the good wettability of the formulation. More precisely, the first phase of mucoadhesion was initiated by chain interdiffusion, which was caused by hydrogen bonds and electrostatic interactions between the polymers and the residual mucoglycoproteins [28,58]. Thereby, the gastric mucoglycoproteins, mainly MUC5AC and MUC6, consist of side chains, i.e., *N*-acetylgalactosamine, *N*-acetylglucosamine, galactose, fucose and sialic acid, which interacted with the polymeric fibers in Caricol^®^-Gastro. These include β-glucan from oat flour and α-(1→4)-d-galacturonan from papaya pulp [14,59,60]. The visual observation was confirmed by spectroscopic quantification. Therefore, it can be concluded that the formulation remains on the gastric mucosa long enough to protect the tissue and at the same time to allow the enzymes, i.e., papain and leukopapain, to interact with the ulcerated parts. Leukopapain in particular has been shown in both animal models and clinical settings to promote wound healing on contact with score tissue [16,59]. As a result, a reduction in pain and other gastritis-related symptoms, such as flatulence and constipation, is achieved [14,16].

Organic oat flour at low concentration (oat I) exhibited a different behavior since the viscous modulus was larger than the elastic one over the entire shear range, resulting in a high tan δ value. This indicates that no stable network was formed. The micrographs also showed a delicate network structure interspersed with starch particles with comparatively large pores (highest pore volume percentage 5800 nm). Hence, the network was less stable than Caricol^®^-Gastro and was destroyed by shear stress applied during the rheological measurements. Moreover, the high standard deviations obtained during the measurements can be attributed most likely to the starch particles [52,53]. For oat I the in vitro contact measurements could not be conducted as agglutination of the syringe occurred, which was also because of the starch particles. The ex vivo results are in accordance with the mucin interaction studies carried out via rheological analysis. Oat I was washed away within the first few minutes because of the weakly structured network lacking chain interdiffusion and consequently mucin interaction.

At an increased oat flour concentration (oat II), the ratio of the viscoelastic moduli shifted, more specifically, the elastic modulus was larger than the viscous modulus resulting in an increased network stability. The network became tighter and the pore size distribution shifted towards smaller values (highest volume parentage: 3600–4800 nm). However, the observed network still seemed less stable than that of Caricol^®^-Gastro. When mixed with SGF the network did not change. The adhesive studies showed that a higher adhesion to mucins was observed than for oat I, but the interactions were significantly (*p* < 0.01) weaker than in Caricol^®^-Gastro. Again, spreadability could not be confirmed in vitro because of agglutination of the syringe. The ex vivo studies showed that due to the stable viscoelastic network comparable results to Caricol^®^-Gastro were obtained after 10 min. However, after 30 min the network loosened and adhesion decreased.

Our results show that the mucoadhesive behavior of Caricol^®^-Gastro is not only due to starch (β-glycan) in oat, but also to the fact that other components, especially α-(1→ 4)-d-galacturonan from papaya pulp, contribute to the strengthening of the network. Consequently α-(1→ 4)-d-galacturonan increases the binding sites to the underlying tissue resulting in increased wettability and adhesion [60]. Adhesive interactions are usually triggered by hydrogen bonds as well as electrostatic and hydrophobic forces. Studies on pectin, with α-(1→4)-d-galacturonan as main polymer compound, show that the fibers have excellent wetting abilities and can both mix and interpenetrate with mucin chains [58]. According to literature most of the pectin in papaya consists of a smooth backbone structured by homogalacturonan regions [61]. The increased adhesion of Caricol^®^-Gastro due to its polymeric compounds, i.e., β-glycan of oat and α-(1→4)-d-galacturonan of pectin, was clearly shown in the results of the mucin interaction studies and ex vivo investigations. While the network formation and interactions for β-glycan in oat can be increased via polymeric concentration, the mucin interaction of the individual compounds is comparatively weak [57,58,62].

## 5. Conclusions

The data obtained make it clear that studies on mucoadhesion require a number of methods that need to be coordinated in order to obtain meaningful data. By performing rheological investigations, visualization of the network via cryo-SEM, rheological studies regarding mucin interactions, in vitro wettability, and ex vivo adhesion studies, considering quantification of the material that remains on excised gastric mucosa, a sound understanding of how Caricol^®^-Gastro behaves in the stomach could be established. Caricol^®^-Gastro forms a coherent and stable viscoelastic gel. It exhibits shear thinning properties, spreads on the gastric epithelium, and adheres to the gastric mucus for a certain period of time independent of residual dilution with gastric acid and the motility of the stomach. Thereby, a sufficient high concentration of organic oat rather supports the network formation and only to a lesser extent the adhesion. It is suggested that components of papaya pulp, such as α-(1→4)-d-galacturonan further strengthen the network, increase interactions with mucins and interpenetration with the mucin chains, resulting in prolonged residence time. The in vitro results correlate with the in vivo clinical data. This shows that the coordinated in vitro test combination can be used in the future to investigate mucosal adhesion of dosage forms and also allows a deeper understanding of the adhesive mechanisms in a cost-effective and time-saving manner. By exchanging the excised tissue for another epithelium (e.g., oral mucosa) and adjusting the physiological conditions (e.g., agitation or physiological fluid) one would have a variable system to perform mucoadhesion studies.

## Figures and Tables

**Table 1:** Overview of the ingredients of Caricol^®^-Gastro.

Caricol^®^-Gastro	Ingredients	Concentration (g)
100 g aqueous gel	Papaya pulp (containing pectin, more specifically α-(1→4)-d-galacturonan main polymeric component) * Oat flour (containing starch and β-glucan as main polymeric component) * Apple juice concentrate * Natural flavoring	39.44/100 g 6.25/100 g 10.5/100 g 0.08/100 g

* obtained from biological origin.

**Table 2:** Overview of the calculations for viscoelastic parameters according to.

Interaction Parameters (Differential)	Formula	Shear Rates
Δη/η	Δη = η_mixture_ * (η_formulation_ + η_mucin_)	0.1 s^−1^ (low), 5.18 s^−1^ (medium), 100 s^−1^ (high)
ΔG’/G’	ΔG’ = G’_mixture_ − (G’_formulation_ + G_mucin_)	
Δtan δ	Δtan δ = tan δ_mixture_ – (tan δ_formulation_ + tan δ_mucin_)	

All rheological measurements were carried out in triplicates (*n* = 3).

**Table 3:** Overview of contact angles obtained in horizontal and inclined position on adenocarcinoma (AGS)-cell layer surfaces.

Formulation	Contact Angle Horizontal	Contact Angle Inclined (35 °C)
Caricol^®^-Gastro mixed with 0.1 M HCl (1:1)	38° ± 5°	28° ± 1° **
Oat I, II	Measurement not possible due to agglutination in the syringe and, consequently, no drop formation possible.	
MQ-water mixed with 0.1 M HCl (1:1)	101° ± 2°	70° ± 2° ***

** (*p* < 0.01), *** (*p* < 0.001).
